# Follistatin-like 1 (FSTL1) is a prognostic biomarker and correlated with immune cell infiltration in gastric cancer

**DOI:** 10.1186/s12957-020-02070-9

**Published:** 2020-12-08

**Authors:** Li Li, Shanshan Huang, Yangyang Yao, Jun Chen, Junhe Li, Xiaojun Xiang, Jun Deng, Jianping Xiong

**Affiliations:** 1 Department of Oncology, The First Affiliated Hospital of Nanchang University, Nanchang, Jiangxi P.R. China; 2 Jiangxi Key Laboratory for Individualized Cancer Therapy, Nanchang, Jiangxi P.R. China

**Keywords:** FSTL1, Tumor immune infiltrates, Prognostic biomarker, Gastric cancer

## Abstract

**Background:**

Follistatin-like 1 (FSTL1) plays a central role in the progression of tumor and tumor immunity. However, the effect of FSTL1 on the prognosis and immune infiltration of gastric cancer (GC) remains to be elucidated.

**Methods:**

The expression of FSTL1 data was analyzed in Oncomine and TIMER databases. Analyses of clinical parameters and survival data were conducted by Kaplan-Meier plotter and immunohistochemistry. Western blot assay and real-time quantitative PCR (RT-qPCR) were used to analyze protein and mRNA expression, respectively. The correlations between FSTL1 and cancer immune infiltrates were analyzed by Tumor Immune Estimation Resource (TIME), Gene Expression Profiling Interactive Analysis (GEPIA), and LinkedOmics database.

**Results:**

The expression of FSTL1 was significantly higher in GC tissues than in normal tissues, and bioinformatic analysis and immunohistochemistry (IHC) indicated that high FSTL1 expression significantly correlated with poor prognosis in GC. Moreover, FSTL1 was predicted as an independent prognostic factor in GC patients. Bioinformatics analysis results suggested that FSTL1 mainly involved in tumor progression and tumor immunity. And significant correlations were found between FSTL1 expression and immune cell infiltration in GC.

**Conclusions:**

The study effectively revealed useful information about FSTL1 expression, prognostic values, potential functional networks, and impact of tumor immune infiltration in GC. In summary, FSTL1 can be used as a biomarker for prognosis and evaluating immune cell infiltration in GC.

**Supplementary Information:**

The online version contains supplementary material available at 10.1186/s12957-020-02070-9.

## Background

Gastric cancer (GC) is the fourth most common cancer worldwide; despite the rapid development of diagnosis and therapies, GC is still one of the most leading cause of death [1]. Thus, it is urgent to investigate the mechanism of cancer progression, as well as explore the potential biomarkers of diagnosis, prognosis, and therapy in GC. In recent years, immunotherapy has rapidly developed into a promising and effective therapeutic strategy for GC patients [2]; however, the molecular mechanisms related to immune dysfunction remain unknown. Several studies have uncovered significant correlations between tumor microenvironment (TME) and the mechanisms of immune dysfunction [3–5]. Tumor-infiltrating lymphocytes (TILs), including tumor-associated macrophages (TAMs) and tumor-infiltrating neutrophils (TINs), display unique histological features that can distinguish the differentiation of malignancies and often vary between individual patients. In addition, TILs have been proven to play essential roles in the prognosis and treatment of malignancies [6–9]. Hence, it is important to clarify the immunophenotype of tumor-immune interactions, as this will lead to the identification of novel immunotherapeutic-related targets in various malignancies.

Follistatin-like 1 (FSTL1) is a transmembrane extracellular glycoprotein of the secreted protein acid and belongs to the BM-40/SPARC/osteonectin family. FSTL1 was first cloned in 1988 from the synovial tissues of patients with rheumatoid arthritis after it was detected in the serum and synovial fluid of patients with the disease [10]. Immunological studies suggest that FSTL1 regulates joint inflammation in arthritic diseases, while also modulating allograft tolerance. In addition, FSTL1 acts as an autoantigen in patients with rheumatoid arthritis by inhibiting IL6 and producing IL17 to play cardioprotective roles in several cardiovascular diseases [10, 11]. In previous reports, FSTL1 has been shown to produce varying effects in terms of tumors progression and inhibition. For example, FSTL1 has been found to promote tumor inhibition in breast cancer [12] and clear-cell renal cell carcinoma [13]; however, it is a poor prognostic indicator of colorectal cancer [14], esophageal squamous cell carcinoma [15], and hepatocellular carcinoma [16]. FSTL1 also affects the progression of multiple tumors by modulating the host immune system, leading to immune dysfunction in many cases. FSTL1-induced immune dysfunction leads to tumor bone metastases [17] and activation of immune evasion mechanisms in patients with non-small cell lung cancer (NSCLC) through the FSTL1-DIP2A axis [18]. FSTL1 plays a crucial role in tumor-immune and cancer cell progression. However, the effects of FSTL1 on GC prognosis and immune infiltration are still unclear. The potential functions and mechanisms of FSTL1 in cancer progression and immunology need to be discovered.

In this study, we investigated the expression of FSTL1 in GC using bioinformatics analysis, as well as clinical samples, and analyzed the association of FSTL1 expression with different TILs in TME using the Tumor Immune Estimation Resource (TIMER) database and CIBERSORT method. The results demonstrate that FSTL1 holds prognostic value, and explain the potential mechanism and impact of FSTL1 and TME in GC.

## Methods

### Bioinformatic analysis

FSTL1 mRNA expression was assessed in GC using Oncomine V.4.5 (www.oncomine.org), Gene Expression Profiling Interactive Analysis (GEPIA) (http://gepia.cancer-pku.cn/index.html), and TIMER database (https://cistrome.shinyapps.io/timer/) [19–21]. The correlation between FSTL1 expression and survival in GC were analyzed with the Kaplan-Meier plotter [22]. TIMER database was used to investigate FSTL1 expression in multiple cancers and the correction between FSTL1 expression and TME in GC [20, 23]. The GSVA package of R language was used to detect activity changes of immune-related pathway after FSTL1 expression changes [24], and the sources of immune-related pathway were used in Molecular Signatures Database (http://software.broadinstitute.org/gsea/msigdb) [25]. Median FSTL1 expression level of tumors from The Cancer Genome Atlas (TCGA) database (https://cancergenome.nih.gov) was grouped into high and low expression groups. Immune-related pathway predictions in different levels of FSTL1 expression were performed using immune-related signatures. The screening criteria were *p* value < 0.01 and false discovery rate (FDR) < 0.01. For the prediction of tumor-infiltrating lymphocytes (TILs), the expression matrices were uploaded to CIBERSORT (https://cibersort.stanford.edu/) [26]. The valid samples were selected using *p* value < 0.05. Both gene ontology (GO) functional enrichment analysis and Kyoto Encyclopedia of Genes and Genomes (KEGG) pathway enrichment analysis were performed utilizing the Database for Annotation, Visualization and Integrated Discovery (DAVID) v6. 8[27, 28].

### Patients and tissue specimens

All the clinical GC samples were obtained from the First Affiliated Hospital of Nanchang University. Formalin-fixed, paraffin-embedded samples from 240 GC patients were collected from January 2011 to October 2012, and 50 fresh GC tissues and paired adjacent noncancerous tissues were stored in liquid nitrogen before use. Before the operation, the patients had not accepted chemotherapy, radiotherapy, or other treatments. All samples were collected with the consent of the patient, and the study was approved by the Ethics Committee of the First Affiliated Hospital of Nanchang University. All patient specimens and clinical data involved in this study complied with the Declaration of Helsinki.

### RNA extraction and real-time quantitative PCR

Total RNA from GC fresh tissues was extracted by TRIzol reagent (Invitrogen, USA) and reverse transcribed by EasyScript First-Strand cDNA Synthesis SuperMix Kit (TransGen Biotech, China). RT-qPCR was evaluated by the StepOnePlus Real-Time PCR System (Applied Biosystems, USA) and Fast Start Universal SYBR Green Master Mix (Takara, Japan). The relative expression of FSTL1, forward (5′-GCCATGACCTGTGACGGAAA-3′); reverse (5′-CAGCGCTGAAGTGGAGAAGA-3′) relative to the internal control (GAPDH) was analyzed using the 2−ΔΔCT method.

### Western blot

The total protein was extracted with RIPA buffer containing protease inhibitor cocktail, separated with 10% SDS-PAGE gel and transferred to polyvinylidene difluoride membrane (Bio-Rad Laboratories, USA). And the membranes were blocked by 10% milk solution for 1 h at room temperature following incubated with primary antibodies against FSTL1 (1:1000, DF12274, Affinity, China) and β-actin (1:1000; #4970; Cell Signaling Technology, USA) for overnight at 4 °C. Then the membranes were washed 3 times in Tri-buffered saline with Tween-20, and incubated with horseradish peroxidase (HRP)-conjugated secondary antibodies (Anti-rabbit IgG, #7074, Cell Signaling Technology, USA) for 1 h at room temperature. After washed 3 times in Tri-buffered saline with Tween-20, the signals were detected with super sensitive regent (Thermo Fisher Scientific, USA).

### Immunohistochemistry

Formalin-fixed paraffin-embedded tissues sections (4-μm) were degreased in xylene and rehydrated in different concentrations of alcohol and distilled water before antigen retrieval. The sections were blocked in 3% H_2_O_2_ at room temperature for 5–10 min to eliminate endogenous peroxidase activity. Then sections were incubated with anti-FSTL1 antibody (1:100, DF12274, Affinity, China) at 4 °C overnight. The slides were washed 2 times in PBS for 5 min each time and incubated with secondary antibodies for 40 min at room temperature, then washed 3 times in PBS. 3,3′-Diaminobenzidine and hematoxylin were used to color these slides. FSTL1 expression level was scored according to the proportion of stained tumor cells and the intensity of the staining [29]. The staining intensity was scored as: 0 (negative), 1 (weak), 2 (moderate), and 3 (strong). The proportion of staining was scored as: 0 (0–5%); 1 (5–25%); 2 (25–50%); 3 (50–75%); and 4 (75–100%). The final FSTL1 expression scores were calculated by multiplying the above two scores. Slides were considered as low or high expression, with final scores of ≤ 6 or > 6, respectively. Two independent pathologists who were blinded observed the results under an optical microscope.

### Statistical analysis

The survival curves of Kaplan-Meier plots and GEPIA databases were displayed by HR with *p* values or Cox *p* values from the log-rank test. Gene expression distribution from the TIMER database was displayed using box plots, with statistical significance assessed by the Wilcoxon test, and the correlation of gene expression from the TIMER databases was evaluated with Spearman’s correlation coefficient and estimated using statistical significance. Kaplan-Meier method and log-rank test were used to analyze overall survival curves. Differences in the mRNA expression levels of FSTL1 in fresh GC and matched normal tissues were analyzed using paired *t* tests. Univariate and multivariate Cox’s proportional hazard models were conducted to analyze the value of each clinic parameter for predicting overall survival. Statistical significance was defined as *p* < 0.05.

## Results

### FSTL1 expression and potential prognosis in GC

**Fig. 1:**
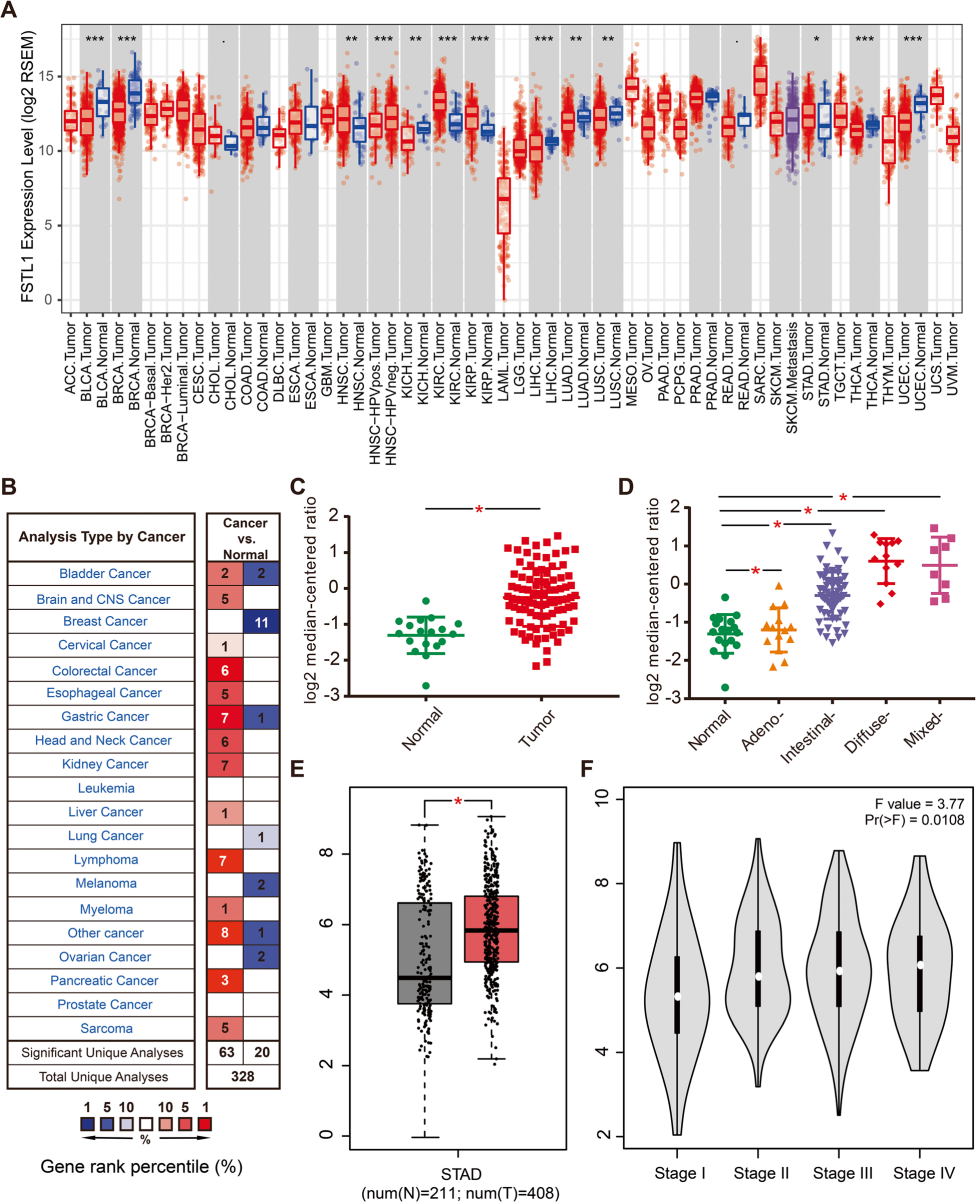
FSTL1 expression data in GC by bioinformatics analysis. **a** FSTL1 expression data in multiple cancer types from TCGA database, as determined by the TIMER database. **b** FSTL1 expression data in multiple cancer types from the Oncomine database. **c**, **d** FSTL1 expression data in Chen Gastric dataset (*N* = 35) in Oncomine. **e**, **f** FSTL1 expression data in GEPIA. (*p < 0.05, **p < 0.01, ***p < 0.001). *FSTL1* follistatin-like 1, *STAD* stomach adenocarcinoma

**Fig. 2:**
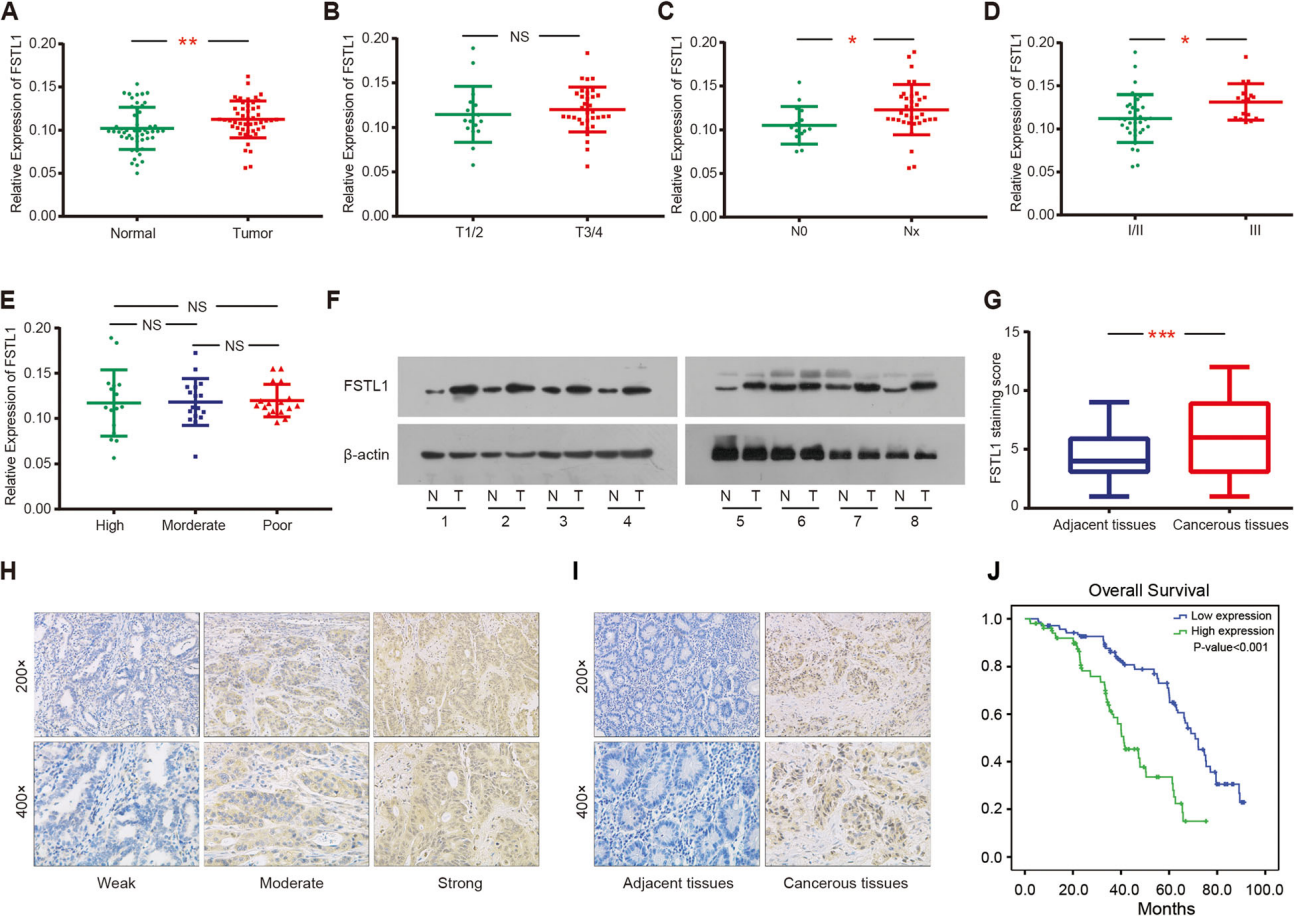
FSTL1 was overexpressed in GC tissues and associated with worse prognosis. **a**–**e** Relationship between FSTL1 mRNA expression and tumor stage or differentiation. **f** The protein expression of FSTL1 in GC patients. **g** The FSTL1 staining score in GC patients. **h** The expression pattern of FSTL1 in GC patients analyzed by immunohistochemistry (IHC). **i** Representative FSTL1-positive/negative staining in cancerous and adjacent normal tissues. **j** The overall survival curve of FSTL1+/− in GC patients. *FSTL1* follistatin-like 1, *N0* GC patients with no lymph node metastasis, *Nx* GC patients with lymph node metastasis, *N* normal, *T* tumor

TIMER and Oncomine databases were used to assess the differential expression of FSTL1 based on cancer type. The majority of data sets from the TIMER and Oncomine database showed that FSTL1 was highly expressed in most malignancies, especially in GC (Fig. 1a, b). In representative Chen Gastric data sets (*N* = 35) from the Oncomine database [30], FSTL1 expression was significantly high expression in GC tissues compared with normal tissues, and was elevated in multiple pathological typing, including adenocarcinoma, intestinal carcinoma, diffuse carcinoma, and mixed carcinoma (Fig. 1c, d). The same result was confirmed in GEPIA database, and the expression of FSTL1 increases with the stage of GC (Fig. 1e, f). In addition, we further confirmed that FSTL1 was highly expressed in GC through real-time quantitative PCR (RT-qPCR) and Western blot (Fig. 2a, f). Moreover, the mRNA expression of FSTL1 in the Nx group (*P* = 0.037) and stage III group (*P* = 0.018) was significantly higher than N0 group and the I/II group in GC patients, respectively (Fig. 2c, d). But there was no significant change in FSTL1 expression in stage T grouping and differentiation grouping (Fig. 2b, e).

Meanwhile, KM-plotter and GEPIA database survival analysis indicated that high FSTL1 expression indicated poor prognosis in GC patients (Supplementary Figure 1A, B). To confirm the predicted prognostic value of FSTL1 in GC, FSTL1 were analyzed in random selected 240 primary GC tissues and paired adjacent non-tumor tissues by immunohistochemistry. The staining of FSTL1 protein ranged from weak to strong (Fig. 2h), and the result showed that FSTL1 positive staining was increased in GC tissues than adjacent non-tumor tissues (Fig. 2i). The positive expression rate of FSTL1 (43.3%, 104/240) in GC samples was significantly higher than that in adjacent non-tumor tissues (15%, 36/240) (*P* < 0.001) (Fig. 2g).

Kaplan-Meier analysis and log-rank test were performed to analyze the prognostic value of FSTL1 expression in GC patients. The result showed that patients with positive FSTL1 expression (41.1 ± 3.8 month) had worse overall survival than negative FSTL1 expression (70.9 ± 3.1 month) (*P* < 0.001) (Fig. 2j).

### Correlation of FSTL1 expression and clinical prognosis in GC with different clinicopathological features

To further understand the effect of FSTL1 in the prognosis of GC, we studied the relationship between FSTL1 expression and the clinicopathological features of these cancers using the Kaplan-Meier plotter database and immunohistochemistry assay.

In Kaplan-Meier plotter database, the expression of FSTL1 has a significant correlation with the prognosis of GC patients with various clinicopathological characteristics. It is worth noting that increased FSTL1 expression was associated with worse OS and PFS in stage III–IV and stage N1–3, but not in stage I/II and stage N0 GC patients (Table 1). The result means that FSTL1 expression level can impact the prognosis in the advanced and lymph node metastasis GC patients, which is consistent with the increased expression of FSTL1 in III group and Nx group compared with I/II group and N0 group in Fig. 2c, d.

**Table 1:** Correlation of FSTL1 mRNA expression and clinical prognosis in GC with different clinicopathological factors by Kaplan-Meier plotter

Clinicopathological characteristics	Overall survival (***N*** = 882)			Progression-free survival (***N*** = 646)		
	N	Hazard ratio	***p***-value	N	Hazard ratio	***p***-value
**SEX**						
Female	236	1.89 (1.33-2.7)	**0.00035**	201	1.97 (1.35-2.87)	**0.00036**
Male	545	1.62 (1.31-2.01)	**7.90E-06**	438	1.6 (1.26-2.03)	**9.20E-05**
**STAGE**						
I	67	1.87 (0.69-5.07)	0.21	60	0.47 (0.14-1.57)	0.21
II	140	1.71 (0.94-3.12)	0.075	131	1.6 (0.82-3.12)	0.17
III	305	1.75 (1.31-2.34)	**0.00012**	186	1.94 (1.34-2.81)	**0.00036**
IV	148	1.89 (1.28-2.81)	**0.0013**	141	1.58 (1.05-2.37)	**0.026**
**STAGE T**						
2	241	2.14 (1.4-3.27)	**3.00E-04**	239	1.85 (1.23-2.8)	**0.0029**
3	204	1.72 (1.21-2.45)	**0.0022**	204	1.6 (1.14-2.26)	**0.0063**
4	38	3.17 (1.3-7.71)	**0.0074**	39	4.67 (1.87-11.66)	**0.00035**
**STAGE N**						
0	74	1.75 (0.76-4.06)	0.19	72	1.78 (0.78-4.08)	0.17
1	225	2.34 (1.55-3.53)	**3.10E-05**	222	2.35 (1.59-3.48)	**9.10E-06**
2	121	1.91 (1.22-2.99)	**0.0043**	125	2.06 (1.33-3.18)	**0.00088**
3	76	2.4 (1.4-4.12)	**0.0011**	76	1.96 (1.15-3.34)	**0.012**
1+2+3	422	2.15 (1.66-2.8)	**5.00E-09**	423	2.19 (1.7-2.82)	**5.40E-10**
**STAGE M**						
0	344	2.04 (1.55-2.7)	**2.90E-07**	443	2.08 (1.59-2.71)	**3.30E-08**
1	56	2.05 (1.12-3.75)	**0.018**	56	1.57 (0.85-2.88)	0.14
**LAUREN CLASSIFICATIO**						
Intestinal	320	2.32 (1.69-3.19)	**1.00E-07**	263	2.01 (1.39-2.89)	**0.00014**
Diffuse	241	2.05 (1.45-2.89)	**3.50E-05**	231	2.07 (1.46-2.93)	**2.60E-05**
Mix	32	2.29 (0.82-6.35)	0.1	28	2.71 (0.76-9.64)	0.11
**DIFFERENTIATIONN**						
Poor	165	1.37 (0.92-2.05)	0.12	121	1.42 (0.9-2.25)	0.13
Moderate	67	1.95 (0.99-3.86)	0.051	67	2.32 (1.19-4.52)	**0.011**
Well	32	13.34 (3.03-58.81)	**1.80E-05**	*	*	*
**HER2 STATUS**						
Negative	532	1.63 (1.3-2.04)	**1.70E-05**	408	1.75 (1.35-2.27)	**1.50E-05**
Positive	344	1.67 (1.27-2.19)	**0.00019**	233	1.75 (1.2-2.55)	**0.0034**

*Sample number too low for meaningful analysis; *FSTL1* follistatin-like 1, *GC* gastric cancer

Next, correlation between FSTL1 expression and clinicopathological was investigated (Table 2). The result showed that expression level of FSTL1 was correlated with tumor size (*P* < 0.001), lymph node metastasis (*P* < 0.001), and tumor-node-metastasis (TNM) stage (*P* = 0.001). No significant correlations between FSTL1 expression and ages (*P* = 0.48), gender (*P* = 0.519), depth of invasion (*P* = 0.147), differentiation (*P* = 0.151), nerve invasion (*P* = 0.809), and vascular invasion (*P* = 0.317) were detected. The results are consistent with the results in the KM-Plotter database.

**Table 2:** The association of FSTL1 expression level with clinicopathological characteristics

Variables	Number	FSTL1 expression		***p***-value
		Negative	Positive	
**Ages**				
≤58	116	64	52	0.48
>58	124	74	50	
**Gender**				
Male	164	92	72	0.519
Female	76	46	30	
**Tumor Size**				
<4cm	144	100	44	**<0.001**
≥4cm	96	38	58	
**Depth of invasion**				
T1/T2	84	43	41	0.147
T3/T4	156	95	61	
**Lymph node metastasis**				
Negative	80	62	18	**<0.001**
Positive	160	76	84	
**TNM stage**				
I/II	134	90	44	**0.001**
III	106	48	58	
**Differentiation**				
Low/undifferentiated	145	78	67	0.151
High/moderate	95	60	35	
**Nerve invasion**				
Negative	148	86	62	0.809
Positive	92	52	40	
**Vascular invasion**				
Negative	136	82	54	0.317
Positive	104	56	48	

*TNM* tumor-node-metastasis

Univariate and multivariate analyses were conducted to investigate the independent prognostic factors of GC patients (Table 3). Univariate analysis indicated that tumor size (*P* < 0.001), depth of invasion (*P* < 0.001), lymph node metastasis (*P* < 0.001), TNM stage (*P* < 0.001), vascular invasion (*P* = 0.003), and FSTL1 expression (*P* < 0.001) were significantly correlated with OS of GC patients. These factors were subjected to multivariate analysis, which indicated that tumor size (*P* = 0.006), depth of invasion (*P* = 0.007), lymph node metastasis (*P* < 0.001), TNM stage (*P* < 0.001), and FSTL1 expression (*P* = 0.002) were independent prognostic factors in GC patients.

**Table 3:** Univariate and multivariate analyses of prognostic factors correlated with OS

Variables	Univariate analyses		Multivariate analyses	
	HR (95% CI)	*p*-value	HR (95% CI)	*p*-value
Age (≥57 vs. <57)	1.051 (0.748-1.494)	0.754		
Gender (Male vs. Female)	1.359 (0.948-1.948)	0.095		
Tumor size (≥4cm vs. <4cm)	2.045 (1.442-2.901)	<0.001	1.724 (1.173-2.532)	0.006
Depth of invasion (T3/T4 vs. T1/T2)	3.095 (1.947-4.922)	<0.001	1.935 (1.198-3.128)	0.007
Lymph node metastasis (Positive vs. Negative)	3.885 (2.497-6.044)	<0.001	2.516 (1.585-3.993)	<0.001
TNM stage (III vs. I/II)	6.978 (4.694-10.373)	<0.001	5.077 (3.345-7.706)	<0.001
Differentiation (Low/undifferentiated vs. High/moderate)	0.898 (0.635-1.272)	0.546		
Nerve invasion (Positive vs. Negative)	0.96 (0.671-1.375)	0.825		
Vascular invasion (Positive vs. Negative)	1.678 (1.186-2.374)	0.003	1.328 (0.916-1.925)	0.134
FSTL1 expression (Positive vs. Negative)	3.041 (2.067-4.474)	<0.001	1.973 (1.284-3.031)	0.002

*OS* overall survival, *HR* hazard ratio, *TNM* tumor-node-metastasis, *HR* Hazard ratio

### Protein-protein interaction of FSTL1 network analysis

**Fig. 3:**
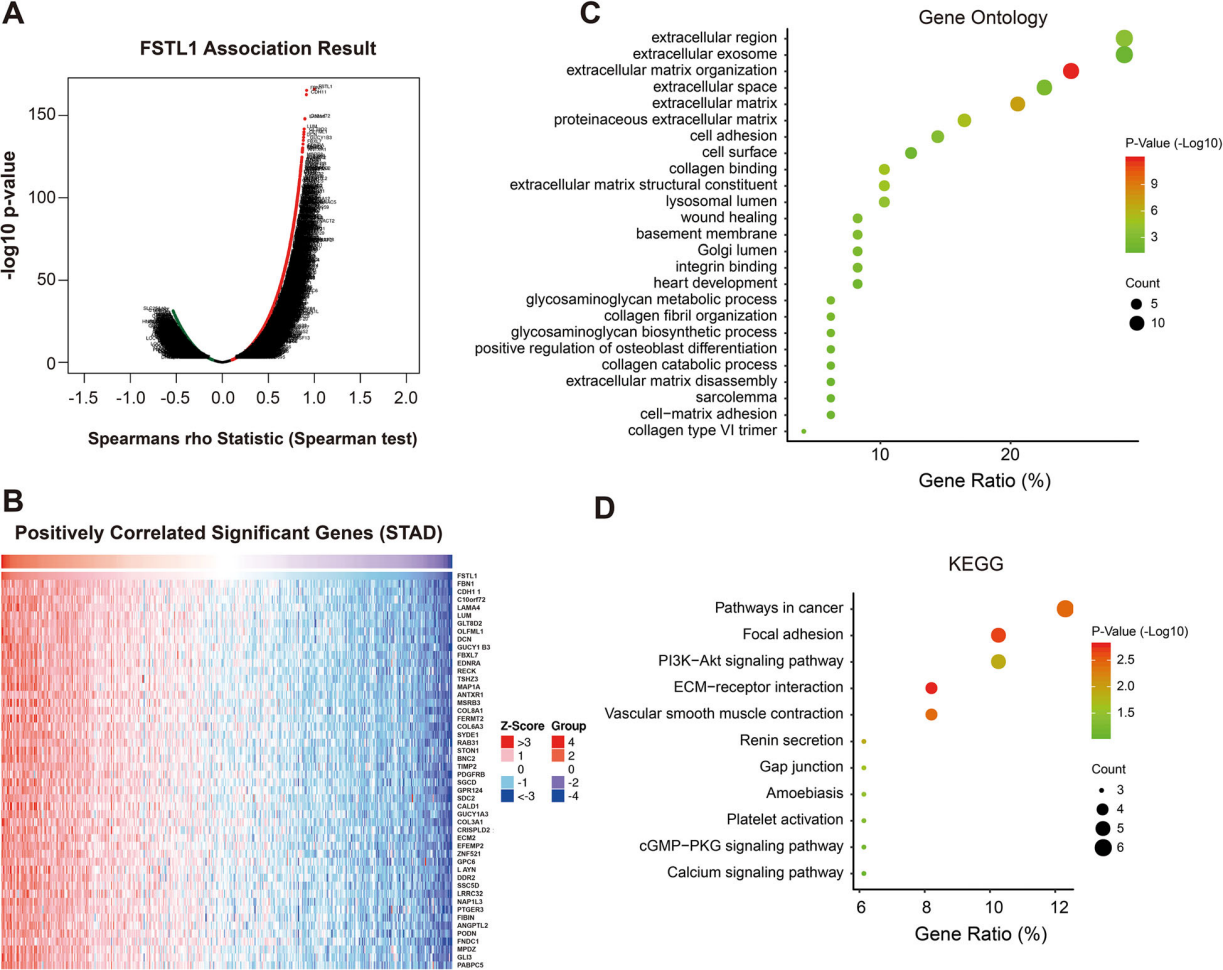
Differentially expressed genes correlation with FSTL1 in GC (LinkedOmics). **a** Correlations between FSTL1 and genes differentially expressed in GC. **b** Heat maps of the most 50 significant genes positively correlated with FSTL1 in GC. **c**, **d** The significantly enriched GO annotations (cellular components, biological processes, molecular functions) and KEGG pathway of FSTL1 50 most positive co-expression genes in GC. *FSTL1* follistatin-like 1, *STAD* stomach adenocarcinoma, *KEGG* Kyoto Encyclopedia of Genes and Genomes

Genes co-expressed with FSTL1 were analyzed by LinkedOmics database, which analyzed mRNA sequencing data of 415 GC patients from TCGA. All the genes associated with FSTL1 GC were showed in Fig. 3a. And Fig. 3b revealed the most 50 significant genes positively correlated with FSTL1 in GC. The 50 genes differentially expressed associated with highly FSTL1 expression were primarily enriched in extracellular region/exosome/space/matrix/matrix organization, cell adhesion (Fig. 3c). KEGG pathway analysis found enrichment in pathway in cancer, focal adhesion, PI3K-Akt signaling pathway, and ECM-receptor interaction, which indicated that elevation of FSTL1 was significantly correlated with tumor development and immune response (Fig. 3d).

**Fig. 4:**
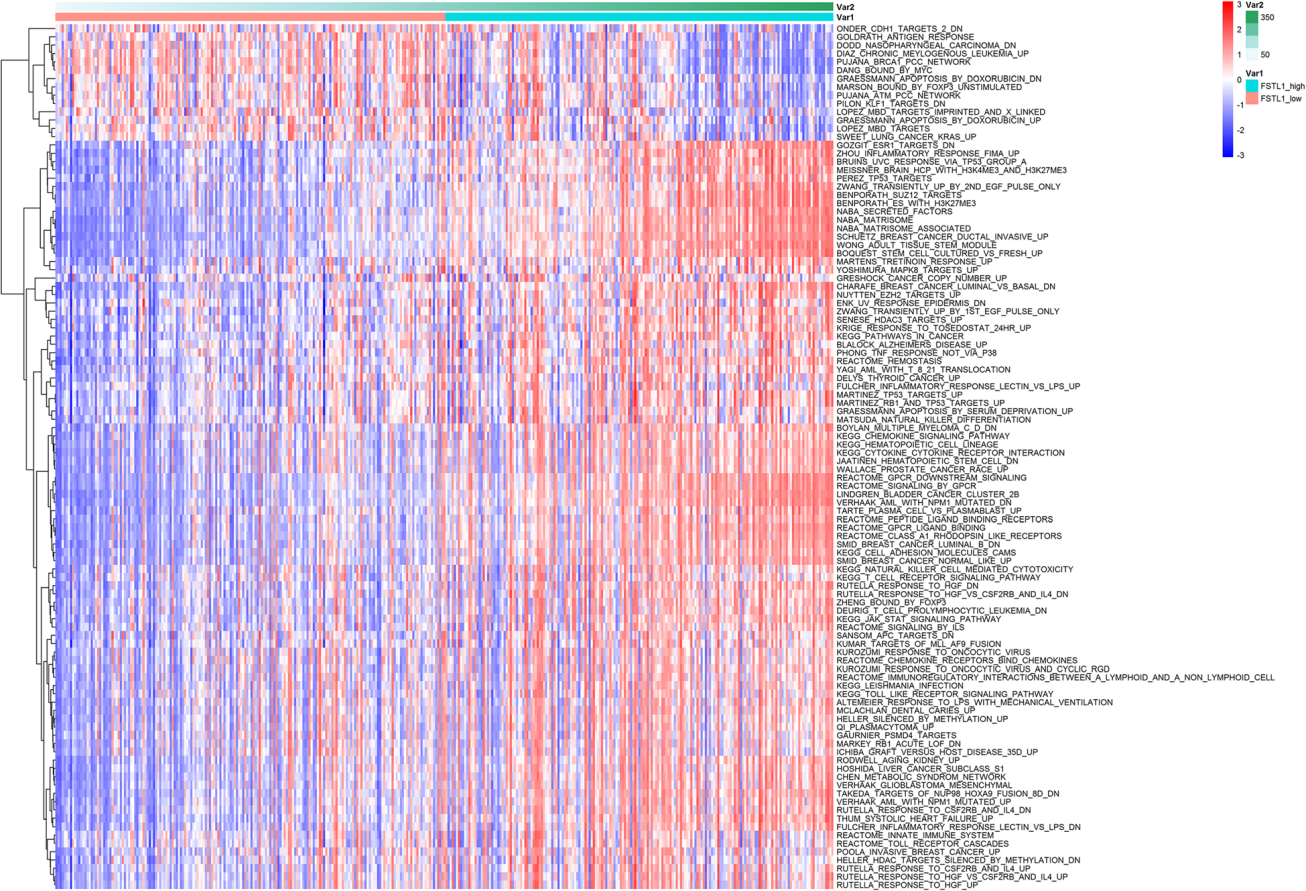
FSTL1 correlated with tumor immunity in GC. GSVA showed immune-related signaling pathways that were significantly activated based on FSTL1 expression levels from TCGA dataset in GC. Var1: the group of FSTL1 expression; Var2: the expression score of FSTL1 expression

In addition, we applied GSVA and clustering on a set of immune-specific signatures to further validate the enrichment of FSTL1 expression in the immune-related pathway. As FSTL1 expression increased, most immune-related pathways were activated in GC, CYTOKINE_CYTOKINE_RECEPTOR_INTERACTION, INNATE_IMMUNE_SYSTEM, NATURAL_KILLER_CELL_MEDIATED_CYTOTOXICITY, and others in stomach adenocarcinoma (STAD) (Fig. 4). Hence, FSTL1 plays a crucial role in immune cell infiltration and tumor-immune system interactions in GC.

### Correlation between FSTL1 and tumor environment in GC

**Fig. 5:**
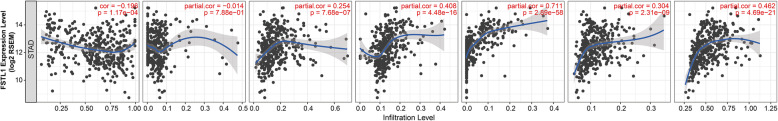
Correlation between FSTL1 expression and the number of immune cell infiltrates in GC. FSTL1 expression is negatively correlated with tumor purity and positively correlated with infiltrating levels of CD8^+^ T cells, CD4^+^ T cells, macrophages, neutrophils, and DCs but no significant relationship with infiltrating level of B cells in GC (*n* = 415). *FSTL1* follistatin-like 1, *STAD* stomach adenocarcinoma

TILs, which are essential components of the TME, have been shown to reflect the host antitumor immune response and are prognostic factors for cancer [31, 32]. As mentioned, GO analysis revealed that FSTL1 has functions associated with extracellular region/exosome/space/matrix/matrix organization, cell adhesion, while KEGG analysis indicated that FSTL1 involved in the ECM-receptor interaction pathway, PI3K-Akt signaling pathway. It is well-known that remodeling the extracellular matrix (ECM), disordered PI3K-Akt signaling pathway can exert a great influence on the TME [33, 34]. Thus, we analyzed the correlation between FSTL1 expression and TILs in GC using the TIMER database. The result showed that FSTL1 expression was found to be correlated with high immune infiltration of CD8^+^ T cells, CD4^+^ T cells, macrophages, neutrophils, and dendritic cells in GC (Fig. 5).

Immune cells are frequently identified based on the expression of cell surface receptors and intracellular markers. To further assess the relationship between FSTL1 and each tumor-infiltrating immune cell, we analyzed the correlation between FSTL1 expression and immune infiltrating cells, including CD8^+^ T cells, T cells (general), B cells, neutrophils, NK cells and dendritic cells (DCs), monocytes, TAMs, and M1/M2 macrophages, in GC using the TIMER and GEPIA databases. In addition, the functional T cells were also investigated, including T helper type 1 (Th1), Th2, Th17, follicular helper T cell (Tfh), and regulatory T (Treg) cells, as well as exhausted T cells. Even if the correlation was adjusted based on tumor purity, the majority of tumor-infiltrating immune cell markers were positively correlated with FSTL1 expression in GC (Table 4).

**Table 4:** Correlation analysis between FSTL1 and related gene markers of immune cells

Description	Gene markers	None		Purity	
		Cor	***P***	Cor	***P***
CD8+ T cell	CD8A	0.26	**8.45E-08**	0.218	**1.83E-05**
	CD8B	0.146	**2.86E-03**	0.121	**1.82E-02**
T cell (general)	CD3D	0.236	**1.25E-06**	0.179	**4.58E-04**
	CD3E	0.26	**8.57E-08**	0.207	**4.71E-05**
	CD2	0.305	**2.82E-10**	0.262	**2.24E-07**
B cell	CD19	0.265	**4.36E-08**	0.234	**4.03E-06**
	CD79A	0.281	**7.09E-09**	0.232	**4.95E-06**
Monocyte	CD86	0.443	**0.00E+00**	0.408	**1.16E-16**
	CD115 (CSF1R)	0.555	**0.00E+00**	0.524	**3.68E-28**
TAM	CCL2	0.545	**0.00E+00**	0.52	**1.36E-27**
	CD68	0.216	**9.23E-06**	0.178	**5.08E-04**
	IL10	0.446	**1.15E-21**	0.425	**4.09E-18**
M1 Macrophage	INOS (NOS2)	0.007	8.88E-01	-0.002	9.65E-01
	IRF5	0.158	**1.22E-03**	0.129	**1.17E-02**
M2 Macrophage	CD163	0.492	**0.00E+00**	0.461	**2.46E-21**
	VSIG4	0.53	**0.00E+00**	0.516	**4.01E-27**
	MS4A4A	0.544	**0.00E+00**	0.521	**8.69E-28**
Neutrophils	CD66b (CEACAM8)	0.013	**7.85E-01**	0.036	4.80E-01
	CD11b (ITGAM)	0.493	**0.00E+00**	0.472	**2.04E-22**
	CCR7	0.362	**2.81E-14**	0.315	**3.47E-10**
Natural killer cell	KIR2DL1	0.144	**3.38E-03**	0.131	**1.09E-02**
	KIR2DL3	0.12	**1.49E-02**	0.098	5.60E-02
	KIR2DL4	-0.052	2.87E-01	-0.097	5.89E-02
	KIR3DL1	0.131	**7.60E-03**	0.112	**2.86E-02**
	KIR3DL2	0.15	**2.18E-03**	0.129	**1.23E-02**
	KIR3DL3	-0.104	**3.35E-02**	-0.092	7.23E-02
	KIR2DS4	0.054	2.71E-01	0.039	4.45E-01
Dendritic cell	HLA-DPB1	0.278	**1.01E-08**	0.215	**2.40E-05**
	HLA-DQB1	0.137	**5.30E-03**	0.073	1.58E-01
	HLA-DRA	0.198	**5.01E-05**	0.142	**5.55E-03**
	HLA-DPA1	0.233	**1.67E-06**	0.178	**5.05E-04**
	BDCA-1 (CD1C)	0.43	**4.41E-20**	0.4	**5.47E-16**
	BDCA-4 (NRP1)	0.703	**0.00E+00**	0.682	**2.88E-53**
	CD11c (ITGAX)	0.453	**0.00E+00**	0.418	**1.84E-17**
Th1	T-bet (TBX21)	0.235	**1.32E-06**	0.202	**7.32E-05**
	STAT4	0.368	**1.12E-14**	0.337	**1.73E-11**
	STAT1	-0.018	7.18E-01	-0.044	3.95E-01
	IFN-γ (IFNG)	-0.012	8.09E-01	-0.038	4.61E-01
	TNF-α (TNF)	0.116	**1.78E-02**	0.069	1.82E-01
Th2	GATA3	0.309	**1.66E-10**	0.287	**1.27E-08**
	STAT6	0.052	2.86E-01	0.047	3.59E-01
	STAT5A	0.362	**3.79E-14**	0.343	**6.92E-12**
	IL13	0.13	**8.14E-03**	0.149	**3.56E-03**
Tfh	BCL6	0.447	**0.00E+00**	0.412	**6.29E-17**
	IL21	0.1	**4.19E-02**	0.082	1.13E-01
Th17	STAT3	0.402	**0.00E+00**	0.38	**1.96E-14**
	IL17A	-0.153	**1.76E-03**	-0.17	**8.82E-04**
Treg	FOXP3	0.273	**1.84E-08**	0.229	**6.89E-06**
	CCR8	0.395	**5.70E-17**	0.379	**2.26E-14**
	STAT5B	0.542	**0.00E+00**	0.533	**3.02E-29**
	TGFβ (TGFB1)	0.565	**0.00E+00**	0.539	**6.66E-30**
T cell exhaustion	PD-1 (PDCD1)	0.147	**2.78E-03**	0.104	**4.30E-02**
	CTLA4	0.157	**1.35E-03**	0.117	**2.23E-02**
	LAG3	0.121	**1.39E-02**	0.079	1.26E-01
	TIM-3 (HAVCR2)	0.427	**0.00E+00**	0.399	**7.03E-16**
	GZMB	0.056	2.54E-01	-0.004	9.36E-01

*TAM* tumor-associated macrophage, *Th* T helper cell, *Tfh* Follicular helper T cell, *Treg* regulatory T cell, *Cor* R value of Spearman’s correlation, *None* correlation without adjustment, *Purity* correlation adjusted by purity

### Correlation between FSTL1 expression and macrophage polarization in GC

**Fig. 6:**
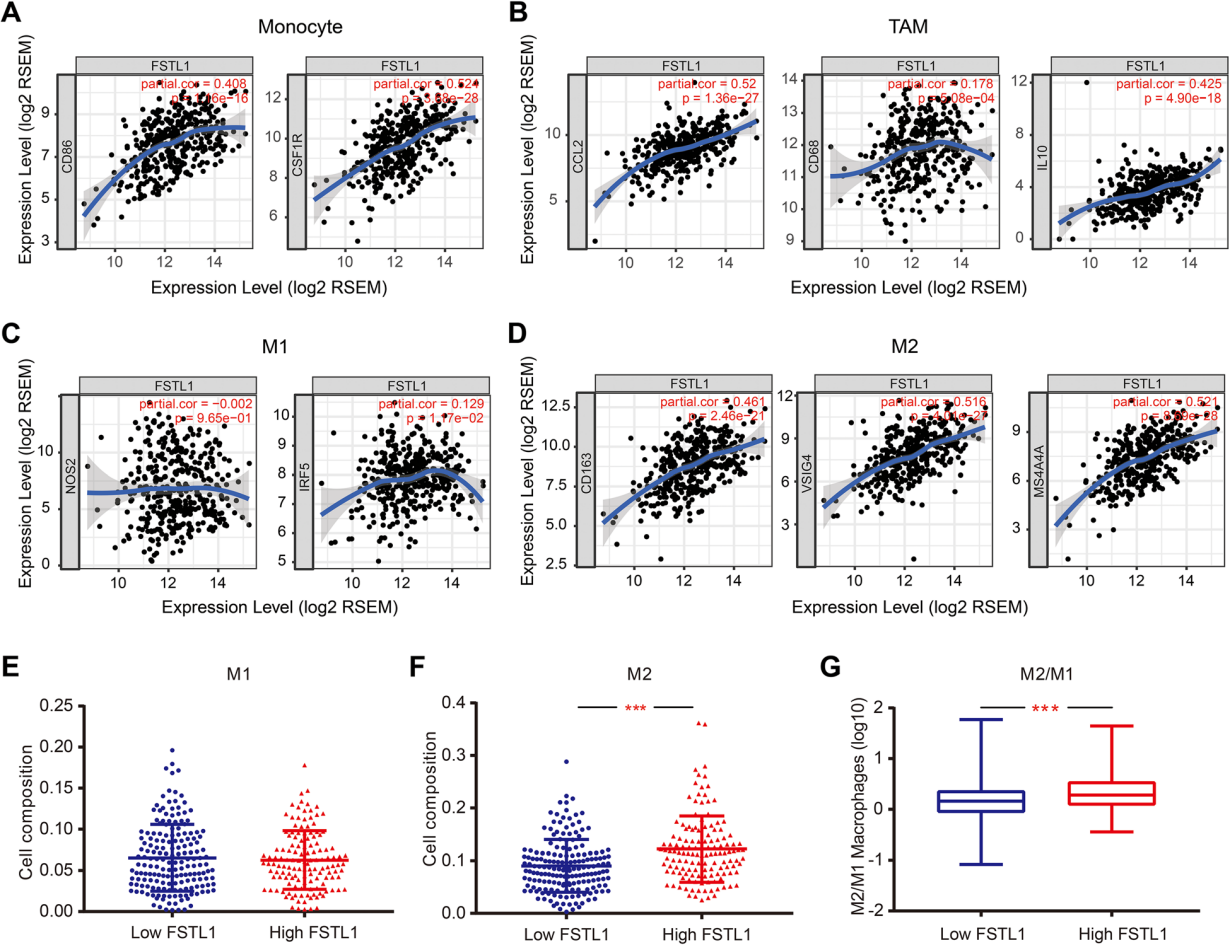
FSTL1 expression is related to macrophage polarization in GC. **a**–**d** FSTL1 expression is significantly correlated with the genetic markers for monocytes (CD86, CSF1R), TAM (CCL2, CD68, IL10), M1 (NOS2, IRF2), and M2 monocytes (CD163, VSIG4, MS4A4A) in GC (*n* = 415). **e**, **f** Relative density of M1 and M2 macrophages in FSTL1 high and low expression groups. **g** Ratio of M2 to M1 macrophages in FSTL1 high and low expression group. *FSTL1* follistatin-like 1, *TAM* tumor-associated macrophage

Studies have shown that polarization from the antitumor M1 (classically activated) macrophage to protumor M2 (alternatively activated) macrophage phenotype is correlated with tumor development [35, 36]. Interesting, we found that the markers for monocytes, TAMs, and M2 macrophages showed moderate-to-strong correlations with FSTL1 expression, while the M1 macrophage markers were weakly correlated with FSTL1 expression (Fig. 6a–d, Table 4). To confirm the results with the TIMER database, the correlation between FSTL1 expression and the cell markers for monocytes, TAMs, M1 macrophages, and M2 macrophages in GC was assessed using GEPIA database. Findings from the GEPIA database were consistent with the TIMER database (Supplementary Table 1). Therefore, we used the CIBERSORT method to further explore whether FSTL1 expression was correlated with the polarization of macrophages in GC. The results showed that high FSTL1 expression had a higher ratio of M2 macrophage (Fig. 6e–g).

## Discussion

In this study, we found that (1) FSTL1 was highly expressed and correlated with worse prognosis in GC patients, (2) high expression of FSTL1 was positively correlated with immune infiltration in GC, and (3) high expression of FSTL1 promotes the polarization of M1 macrophages to M2 macrophages in GC.

Studies have showed that FSTL1 was involved in tumor development and tumor immunity by a variety of mechanisms, such as promoting metastasis and chemoresistance through NFκB-BMP signaling pathway in esophageal squamous cell carcinoma [15], activating the focal adhesion signaling pathway to promotes colorectal cancer [14], and causing immune dysfunction in lung cancer [18] and tumor with bone metastasis [37]. From these results, we can speculate that FSTL1 is crucially involved in tumor progression and tumor immunity.

In the present study, we found that FSTL1 expression is upregulated and indicated worse prognosis in GC using bioinformatics. And the results were confirmed by RT-qPCR, Western blot, and immunohistochemistry (IHC) assay. Moreover, the results indicated that high expression level of FSTL1 was correlated with tumor size, lymph node metastasis, and TNM stage, especially. The univariate and multivariate analysis of GC patients suggested that FSTL1 was an independent factor of prognosis in GC. All of the results suggested that FSTL1 may become a potential prognosis predictor in GC patients. To investigate the underlying mechanism, GO and KEGG analyses were performed in DAVID website. Co-expression genes of FSTL1 were enriched in extracellular region/exosome/space/matrix/matrix organization, cell adhesion, and pathway in cancer, focal adhesion, PI3K-Akt signaling pathway, ECM-receptor interaction, which have been proved correlated with tumor development and tumor immunity. Thus, we further investigated the relationship between FSTL1 expression and tumor immunity.

TME, which constitutes an important part of tumor immunity, is a complex environment containing a mixture of nutrients, chemokines, immune cells, and other critical non-cancerous components [38]. The interaction between tumor cells and TME plays a crucial role in tumor growth, progression, chemotherapy, and radiotherapy resistance [39–41]. Importantly, tumor-infiltrating immune cells have been found to play roles in tumor growth, invasion, and metastasis in various forms of cancer [42]. Another important aspect of this research is the close connection between FSTL1 expression and tumor-infiltrating immune cells in GC. The result from the GSVA analysis and TIMER database suggests that FSTL1 expression is closely related to immune-related pathways and significantly correlated with CD8^+^ T cell, CD4^+^ T cell, macrophages, neutrophils, and DCs in GC (Figs. 4 and 5). Hence, high FSTL1 expression can cause a substantial increase in immune cell tumor infiltration.

Existing research has shown that TAMs, which differentiated from peripheral monocytes, are infiltrating around tumor cells. TAMs secrete a variety of cytokines, which play an important role in tumor occurrence, metastasis, and invasion, under the stimulation of TME. According to the different activation status and function, macrophages can be divided into M1 (classically activated macrophages) macrophage and M2 (alternatively activated macrophages) macrophage. M1 macrophages secrete pro-inflammatory cytokines and chemokines, and present antigens on a full-time basis, participate in the positive immune response, and perform the function of immune surveillance; M2 macrophages have only weak antigen presentation ability and secrete inhibitory cytokines such as IL-10 or TGF-β downregulate the immune response and play an important role in immune regulation [43, 44]. It is well known that polarization from the antitumor M1 (classically activated) macrophage to protumor M2 (alternatively activated) macrophage phenotype is correlated with tumor development, and the proportion of M1 and M2 macrophages in TAM is closely related to the prognosis of tumor. The large number differentiation of M2 macrophage is correlated with worse prognosis, suggesting that the differentiation of macrophages has important guiding significance for the clinical treatment of tumor [36, 45, 46]. To further investigate the relationship between FSTL1 and tumor-infiltrating cells, we analyzed the connection between FSTL1 expression and tumor-infiltrating cell markers. First, we found that FSTL1 expression is significantly correlated with surface markers of monocytes (CD86, CD115), TAMs (CCL2, CD68, IL10), M1 macrophages (INOS, IRF5, COX2), and M2 macrophages (CD163, VSIG4, MS4A4A) (Fig. 6a–d, Table 4, Supplementary Table 1). This indicates that FSTL1 may play an important role in macrophage polarization and affect the progression of GC through macrophage polarization.

In terms of TILs, helper T lymphocytes, including Th1 cells, Th2 cells, Tfh cells, Th17 cells, and Treg cells, are considered to be the major players in tumor immunity [47–49]. The surface marker of helper T lymphocytes (Th1 cells, Th2 cells, Tfh cells, Th17 cells, and Treg cells) shows significant correlations with FSTL1 expression (Table 4). Hence, FSTL1 plays a vital role in regulating the potential mechanisms of T cell function in GC. A previous study suggested that high T-bet expression was previously correlated with better DFS and OS in GC patients [50]. Interestingly, FSTL1 expression is positively correlated with T-bet in GC. This indicates that FSTL1 may affect the prognosis of GC by impacting the expression of T-bet. Treg cells have been reported to stimulate GC progression by secreting TGF-β to help the cancer cells escape immune system recognition, and high levels of TGF-β in the TME further stimulate the differentiation and expansion of Treg cells [51, 52]. Previous studies have suggested that Treg cells may be involved in the development and progression of bladder carcinomas and GC [53, 54]. Our results agree that FSTL1 expression is a positive biomarker for Treg cells, including TGF-β in GC. Hence, Treg cells play an essential role in the survival of patients with GC affected by FSTL1.

Furthermore, our results showed that FSTL1 expression is positively related to the infiltration level of DCs and the markers of DCs in GC (Fig. 5, Table 4), which indicated that FSTL1 has strong correlation with DCs infiltration in GC. However, the effects of tumor-infiltrating DCs in GC remain unclear. Additional studies are needed to determine how FSTL1 regulates DCs in GC.

Significant correlations have also been detected between FSTL1 and T cell exhaustion markers in GC (Table 4). Previous studies reported that Treg cells could reversibly inhibit the cytotoxic T cell function of attacking tumor cells [55]. On the other hand, exhausted T cells co-express a wide range of surface inhibitory molecules, such as PD-1, CTLA4, LAG3, and TIM-3 [56]. In general, the higher the number of inhibitory receptors co-expressed by exhausted T cells, the more severe the T cells exhaustion. Although the expression of a single inhibitory receptor does not indicate T cells exhaustion, the co-expression of several inhibitory receptors is an important feature [57, 58]. From our results, the inhibitory receptors, including PD-1, CTLA4, LAG3, and TIM-3, increased in GC with FSTL1 overexpression (Table 4), suggesting that FSTL1 plays a crucial role in the regulation of T cell exhaustion. Hence, FSTL1 is closely correlated with tumor-infiltrating immune cells and indicates that FSTL1 plays a vital role in the immune escape mechanism in patients with GC. Recently, FSTL1 neutralizing antibodies were developed and found to display excellent efficacy in vitro [59]. Also, CTLA4, PD-1, and programmed death ligand-1 (PD-L1) inhibitors have shown promising results in lung cancer, yet the response was unclear in advanced-stage GC [60, 61]. The combined use of FSTL1 neutralizing antibodies with anti-CTLA-4 or anti-PD-1/PD-L1 inhibitors may produce additive or synergistic effects, thus creating a new treatment option. Of course, part of these results have not been proved or confirmed in our specimens, and further investigation should be conducted in the future.

## Conclusions

In summary, FSTL1 is commonly expressed and possesses significant prognostic value in GC. High FSTL1 expression is associated with high infiltration levels of CD8^+^ T cells, CD4^+^ T cells, macrophages, neutrophils, and DCs in GC, and potentially contributes to the regulation of TAMs, DCs, helper T lymphocytes, and T cell exhaustion. Furthermore, high expression of FSTL1 promotes the polarization of M1 macrophages to M2 macrophages in GC, which may be one of the reasons for the poor prognosis of FSTL1 in GC patients. For these reasons, FSTL1 might be a promising prognostic biomarker for cancer and could provide new ideas for improving tumor immune evasion and immunotherapy in GC.

## Supplementary Information

**Additional file 1:** **Supplementary Figure 1**. The FSTL1 overall survival curves of GC patients in KM-plotter (A) and GEPIA (B) database. FSTL1, follistatin-like 1; OS, overall survival, HR, hazard ratio.

**Additional file 2:** **Supplementary Table 1.** Correlation analysis between FSTL1 and the related gene markers of monocyte and macrophages by GEPIA.

## Data Availability

The data sets during and/or analyzed during the current study are available from the corresponding author on reasonable request

## Abbreviations

FSTL1: Follistatin-like 1
GC: Gastric cancer
TIMER: Tumor Immune Estimation Resource
RT-qPCR: Real-time quantitative PCR
GEPIA: Gene Expression Profiling Interactive Analysis
IHC: Immunohistochemistry
DCs: Dendritic cells
TME: Tumor microenvironment
TILs: Tumor-infiltrating lymphocytes
TINs: Tumor-infiltrating neutrophils
TCGA: The Cancer Genome Atlas
KEGG: Kyoto Encyclopedia of Genes and Genomes
DAVID: Database for Annotation, Visualization and Integrated Discovery
TNM: Tumor-node-metastasis
ACC: Adrenocortical carcinoma
BLCA: Bladder urothelial carcinoma
BRCA: Breast invasive carcinoma
CESC: Cervical squamous cell carcinoma and endocervical adenocarcinoma
CHOL: Cholangio carcinoma
COAD: Colon adenocarcinoma
DLBC: Lymphoid neoplasm diffuse large B cell lymphoma
ESCA: Esophageal carcinoma
GBM: Glioblastoma multiforme
HNSC: Head and neck squamous cell carcinoma
KICH: Kidney chromophobe
KIRC: Kidney renal clear cell carcinoma
KIRP: Kidney renal papillary cell carcinoma
LAML: Acute myeloid leukemia
LGG: Brain lower grade glioma
LIHC: Liver hepatocellular carcinoma
LUAD: Lung adenocarcinoma
LUSC: Lung squamous cell carcinoma
MESO: Mesothelioma
OV: Ovarian serous cystadenocarcinoma
PAAD: Pancreatic adenocarcinoma
PCPG: Pheochromocytoma and paraganglioma
PRAD: Prostate adenocarcinoma
READ: Rectum adenocarcinoma
SARC: Sarcoma
SKCM: Skin cutaneous melanoma
STAD: Stomach adenocarcinoma
TGCT: Testicular germ cell tumors
THCA: Thyroid carcinoma
THYM: Thymoma
UCEC: Uterine corpus endometrial carcinoma
UCS: Uterine carcinosarcoma
UVM: Uveal melanoma
