# Supplementary figures and images for: Follistatin-like 1 (FSTL1) is a prognostic biomarker and correlated with immune cell infiltration in gastric cancer
Source: World J Surg Oncol. 2020 Dec 8;18:324. doi: 10.1186/s12957-020-02070-9 (PMC7724795; doi:10.1186/s12957-020-02070-9)

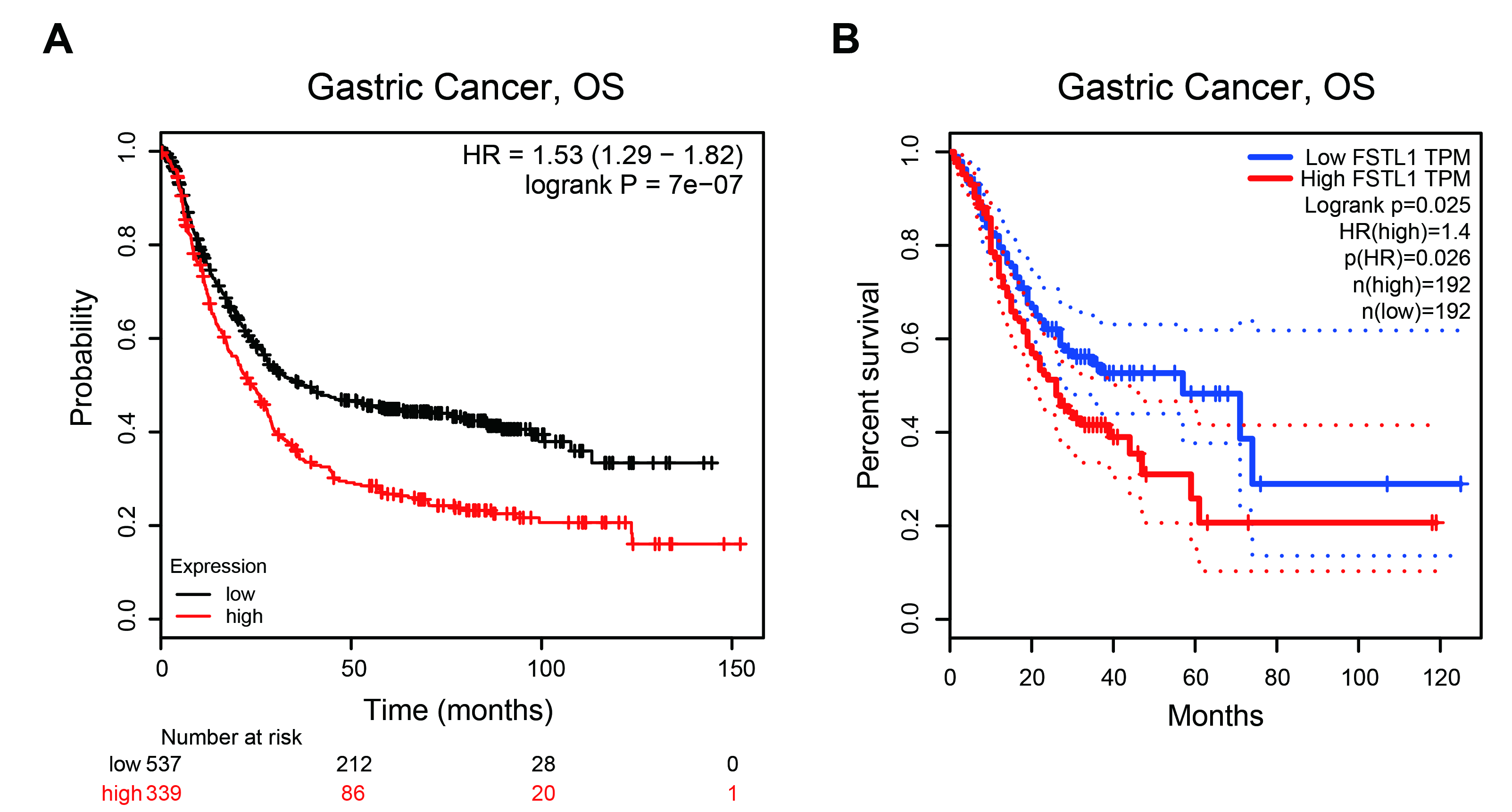

Supplement: Supplementary file 1 — Additional file 1: Supplementary Figure 1. The FSTL1 overall survival curves of GC patients in KM-plotter (A) and GEPIA (B) database. FSTL1, follistatin-like 1; OS, overall survival, HR, hazard ratio. [file 12957_2020_2070_MOESM1_ESM.tif]
