# Supplementary material for: Follistatin-like 1 (FSTL1) is a prognostic biomarker and correlated with immune cell infiltration in gastric cancer
Source: World J Surg Oncol. 2020 Dec 8;18:324. doi: 10.1186/s12957-020-02070-9 (PMC7724795; doi:10.1186/s12957-020-02070-9)
Supplement: Supplementary file 2 — Additional file 2: Supplementary Table 1. Correlation analysis between FSTL1 and the related gene markers of monocyte and macrophages by GEPIA. [file 12957_2020_2070_MOESM2_ESM.docx]

**Supplementary Table 1. Correlation analysis between FSTL1 and related gene markers of monocyte and macrophages in GC.**

| **Description** | **Gene markers** | **R** | | ***p*-value** |
| --- | --- | --- | --- | --- |
| Monocyte | CD86 | 0.51 | **6.50E-29** | |
|  | CD115(CSF1R) | 0.68 | **1.50E-57** | |
| TAM | CCL2 | 0.56 | **9.60E-35** | |
|  | CD68 | 0.39 | **6.00E-16** | |
|  | IL10 | 0.55 | **3.40E-34** | |
| M1 Macrophage | INOS (NOS2) | 0.075 | 0.13 | |
|  | IRF5 | 0.33 | **4.30E-12** | |
| M2 Macrophage | CD163 | 0.52 | **5.00E-30** | |
|  | VSIG4 | 0.59 | **3.40E-40** | |
|  | MS4A4A | 0.62 | **8.70E-45** | |

TAM, Tumor-associated macrophages.
